# Different functions of the common P/V/W and V-specific domains of rinderpest virus V protein in blocking IFN signalling

**DOI:** 10.1099/vir.0.056739-0

**Published:** 2014-01

**Authors:** Senthil K. Chinnakannan, Barbara Holzer, Beatriz Sanz Bernardo, Sambit K. Nanda, Michael D. Baron

**Affiliations:** The Pirbright Institute, Ash Road, Pirbright, Surrey GU24 0NF, UK

## Abstract

The V proteins of paramyxoviruses are composed of two evolutionarily distinct domains, the N-terminal 75 % being common to the viral P, V and W proteins, and not highly conserved between viruses, whilst the remaining 25 % consists of a cysteine-rich V-specific domain, which is conserved across almost all paramyxoviruses. There is evidence supporting a number of different functions of the V proteins of morbilliviruses in blocking the signalling pathways of type I and II IFNs, but it is not clear which domains of V are responsible for which activities and whether all these activities are required for effective blockade of IFN signalling. We have shown here that the two domains of rinderpest virus V protein have distinct functions: the N-terminal domain acted to bind STAT1, whilst the C-terminal V-specific domain interacted with the IFN receptor-associated kinases Jak1 and Tyk2. Effective blockade of IFN signalling required the intact V protein.

## Introduction

It has become clear that many viruses have evolved mechanisms to overcome the immediate innate immune response of the host, usually by encoding one or more proteins that can counteract the induction of IFNs, the signalling pathways by which IFNs exert their action, or the antiviral actions of proteins induced or activated by IFNs (reviewed by Conzelmann, 2005; Haller *et al.*, 2006; Randall & Goodbourn, 2008; Versteeg & García-Sastre, 2010). Viruses in the subfamily *Paramyxovirinae* mediate their IFN evasion activities through the protein products of the P gene, primarily the non-structural proteins V, W and C. The P, V and W proteins are all produced from the P gene by a co-transcriptional editing mechanism, as originally described for measles virus (MV) (Cattaneo *et al.*, 1989), whilst the C proteins, where they exist, are produced from overlapping reading frames within the P gene. The V protein appears to be a particularly important antagonist of both induction and action of IFNs (reviewed by Goodbourn & Randall, 2009; Horvath, 2004; Randall & Goodbourn, 2008), although in viruses of the genus *Respirovirus*, the C protein blocks IFN signalling (reviewed by Fontana *et al.*, 2008; Gotoh *et al.*, 2002; Horvath, 2004).

**Fig. 1.**
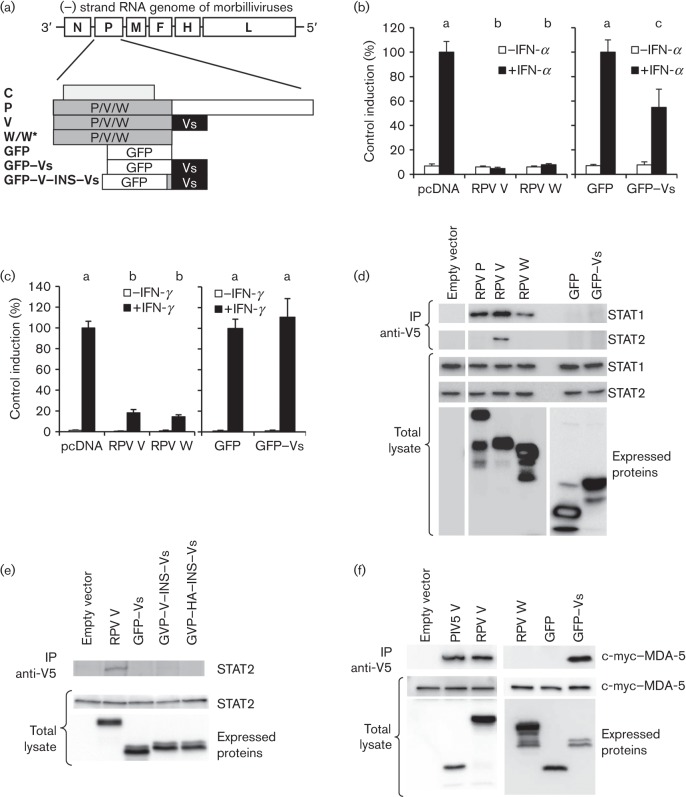
Effects of W and Vs domains on IFN signalling. (a) Protein constructs used in this study and their derivation from the RPV P gene; W*, W_Y110H_. (b, c) Plasmids expressing RPV V, RPV W, GFP or GFP–Vs were transfected into Vero cells together with pJAT-lacZ and either pGL3-MX1P-luc (b) or pGAS-luc (c) as described in Methods. At 24 h post-transfection, the cells were treated with 1000 IU IFN-α ml^−1^ for 8 h (b) or 1000 IU IFN-γ ml^−1^ for 6 h (c). Untreated controls were used in each case. Cells were then lysed, and luciferase and β-galactosidase measured as described in Methods. The results from multiple experiments were normalized to the induction seen in cells transfected with the appropriate control, empty vector (pcDNA) or GFP. Letters above the bars for the IFN-treated samples in (b) and (c) indicate the results of statistical analysis (Tukey analysis for multiple comparisons with maximum probability for significance of *P* = 0.05): results that were not statistically different from one another have the same letter. (d, e) Vero-SLAM cells were transfected with plasmids encoding the indicated constructs or empty vector. The cells were lysed 48 h post-transfection; V5-tagged proteins were immunoprecipitated and STAT1 or STAT2 in the immunoprecipitates (IP) detected by Western blotting. Equal amounts of the total lysates were analysed by Western blotting to detect the expressed proteins and the levels of STAT1 and STAT2 in the cells. (f) 293FT cells were transfected with pCMYC-MDA5 together with plasmids expressing PIV5 V, RPV V, RPV W, GFP or GFP–Vs, or empty vector. Cells were lysed and immunoprecipitation with anti-V5 carried out as in (d). Immunoprecipitates were probed for MDA-5 with anti-c-myc antibody. Equal amounts of the total lysates were analysed by Western blotting to demonstrate the expression of the c-myc-tagged and V5-tagged proteins.

Rinderpest virus (RPV) is a paramyxovirus belonging to the genus *Morbillivirus*, a genus containing several important pathogens, including RPV itself, MV, peste des petits ruminants virus and canine distemper virus (CDV). Each of these viruses is pathogenic in a restricted range of hosts, and studies on the viral proteins of RPV and related morbilliviruses have given insights into the mechanisms that underlie pathogenesis, including the mechanism(s) of host innate immune evasion by these viruses (e.g. Chinnakannan *et al.*, 2013; Devaux *et al.*, 2008; Hahm, 2009; Nanda & Baron, 2006; Pfaller & Conzelmann, 2008; Ramachandran *et al.*, 2008; Rothlisberger *et al.*, 2010). The V proteins of morbilliviruses, as for other paramyxovirus V proteins, are characterized by a highly conserved domain at their C terminus [called the V-specific domain (Vs) in our study], and a longer, less well conserved, N-terminal domain that is common to the P, V and W proteins (Fig. 1a). In our previous studies (Chinnakannan *et al.*, 2013; Nanda & Baron, 2006), we showed that the RPV V protein could block efficiently the signalling pathways of both type I and type II IFNs. We observed that, whilst P, V and W all bound the signal transducer and activator of transcription STAT1, and both V and P blocked the actions of IFN-α and IFN-γ in a reporter gene assay, only the V protein could block effectively IFN-induced phosphorylation of STAT1, whilst P was relatively inefficient (Nanda & Baron, 2006). Subsequently, we showed that the V proteins of RPV and other morbilliviruses could mediate an effective blockade of the type I IFN signalling pathway by means of their ability to interact with multiple components of the IFN signalling pathway, the receptor-associated Janus kinases Jak1 and Tyk2 as well as the signalling molecules STAT1 and STAT2 (Chinnakannan *et al.*, 2013). However, it was not clear what were the roles played individually by the P/V/W shared domain and the Vs domain of the V protein in these various processes, and whether all were necessary for blocking IFN signalling in infected cells. In the present study, we showed that the two domains contribute separately to the blockade of IFN signalling by targeting different components of the Jak–STAT pathway. Importantly, our experiments provided evidence that both activities must be present to fully block IFN signalling in the host cells, as only the entire V protein can fully inhibit IFN action.

## Results and Discussion

To dissect out the relative importance of the P/V/W shared domain (231 aa) and the Vs domain (68 aa) in blocking type I and type II IFN actions, we made plasmid constructs encoding them separately. We based all our P gene derivatives on the virulent Saudi/81 strain of RPV; as the RPV W ORF terminates 2 aa after the editing site, we refer to the P/V/W shared domain as W for brevity. The P, V and W constructs were engineered to prevent C protein expression (Chinnakannan *et al.*, 2013; Nanda & Baron, 2006) and also contained a V5 epitope tag. As the Vs domain by itself is a relatively small peptide, we fused this sequence at its N terminus to GFP to give GFP–Vs; the unmodified GFP served as a negative control for GFP–Vs (Fig. 1a). We studied the ability of V, W and GFP–Vs to block the induction of luciferase from appropriate reporter plasmids: one plasmid with the murine MX1 promoter to report on type I IFN (IFN-α) signalling and one plasmid with a promoter containing multiple copies of the type II IFN (IFN-γ)-responsive γ-activated sequence (GAS). The luciferase reporter assays showed that the W domain could block type I IFN signalling as effectively as the complete V protein; the GFP–Vs construct, however, could only mediate a partial block (Fig. 1b), being ~50 % as effective as V or W. When the same constructs were tested for their ability to block type II IFN action, the W construct again inhibited signalling as well as the full V protein, but the GFP–Vs construct was completely ineffective (Fig. 1c).

As our recent studies suggested that the ability of a morbillivirus V protein to block type II IFN signalling was related to its ability to bind STAT1 (Chinnakannan *et al.*, 2013), we looked at the ability of W and GFP–Vs to bind STAT1 and STAT2, as judged by their ability to co-precipitate these proteins from cells in which the viral proteins were transiently expressed. As we observed previously for the equivalent proteins of the vaccine strain of RPV (Nanda & Baron, 2006), the P, V and W proteins of the virulent strain of RPV all co-precipitated STAT1, whilst only the V protein co-precipitated STAT2 (Fig. 1d). GFP–Vs co-precipitated neither STAT1 nor STAT2 (Fig. 1d). In case the construction of GFP–Vs had omitted an essential amino acid very close to the W/Vs boundary or the Vs domain was being sterically inhibited by the proximity of the GFP, we made two further constructs in which either the 12 aa upstream of Vs in the native V protein were inserted (GFP–V–INS–Vs) or a non-V protein sequence of 12 aa [essentially the haemagglutinin (HA) epitope tag] were similarly inserted (GFP–HA–INS–Vs). Neither of these constructs co-precipitated STAT2 (Fig. 1e).

To check whether the Vs domain was folding properly in the GFP–Vs construct, we assayed its ability to bind the cellular pattern recognition receptor protein MDA-5. It has been shown previously for the V proteins of several paramyxoviruses that they bind MDA-5 through their Vs domain (Andrejeva *et al.*, 2004), and we found that, as expected, RPV V bound MDA-5 as well as the positive control, the V protein of parainfluenzavirus type 5 (PIV5 V); importantly, the GFP–Vs construct bound MDA-5 equally efficiently (Fig. 1f). These data, together with our finding that different morbillivirus V proteins block IFN-γ in proportion to their ability to bind STAT1 (Chinnakannan *et al.*, 2013), suggested that the W domain is able to block the actions of type II IFN because it contains the STAT1 binding site, whilst the Vs domain is unable to block the action of this IFN because it does not interact with STAT1.

**Fig. 2.**
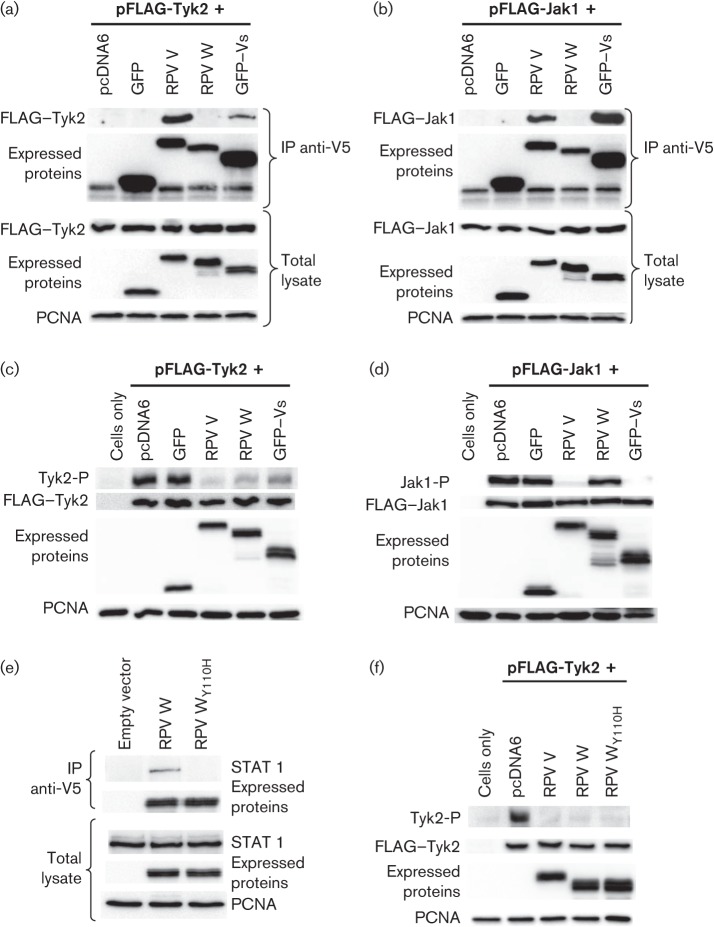
Interaction of components of RPV V with Jak1 and Tyk2. (a, b) 293FT cells were transfected with plasmids encoding FLAG-tagged Tyk2 (a) or FLAG-tagged Jak1 (b) together with plasmids encoding RPV V, RPV W, GFP or GFP–Vs, or empty vector. Cells were lysed 2 days post-transfection and immunoextracted with anti-V5 tag antibody. Immunoprecipitates (IP) were analysed by Western blotting with anti-FLAG or anti-V5 antibodies. Equal amounts of the total lysates were analysed also by Western blotting using anti-FLAG and anti-V5 antibodies. (c, d) Vero-SLAM cells were transfected essentially as in (a) and (b), respectively. At 30 h post-transfection, the cells were lysed in SDS-sample buffer containing phosphatase inhibitors and the levels of phosphoTyk2 (Tyk2-P) (c) or phosphoJak1 (Jak1-P) (d) in the total lysate determined by Western blotting with specific anti-phosphoTyk2 or anti-phosphoJak1 antibodies. Lysates were also analysed to determine the expression levels of individual plasmid-encoded proteins by Western blotting with anti-V5 or anti-FLAG antibodies. (e) Vero-SLAM cells were transfected with plasmids encoding RPV V, W, W_Y110H_ or empty vector. The cells were lysed 48 h post-transfection; V5-tagged proteins were immunoprecipitated and STAT1 in the immunoprecipitates detected by Western blotting. Equal amounts of the total lysates were analysed by Western blotting to detect the expressed proteins and the levels of STAT1 in the cells. (f) Vero-SLAM cells were transfected with FLAG-Tyk2 together with plasmids expressing RPV V, RPV W, RPV W_Y110H_ or empty vector. The cells were lysed and analysed as described (c). Proliferating cell nuclear antigen (PCNA) was used as a loading control throughout.

The observation that the Vs domain is able to block type I IFN action despite binding to neither STAT1 nor STAT2 suggests that this domain acts through a different mechanism. We have shown recently that morbillivirus V proteins interact with the IFN receptor-associated kinases Jak1 and Tyk2, leading to a block of phosphorylation/activation of these enzymes (Chinnakannan *et al.*, 2013). We expressed V, W, GFP and GFP–Vs in 293FT cells along with FLAG-tagged Jak1 or Tyk2, and looked at interactions between these proteins by two assays: (1) the ability of the viral protein to co-precipitate Jak1 or Tyk2, and (2) the ability of the viral protein to block the autophosphorylation observed when either of these kinases is overexpressed, either transiently or as a transgene. This latter activity can be taken as a model of the ability of a protein to block IFN-stimulated activation of the kinase (Chinnakannan *et al.*, 2013; Domanski *et al.*, 1995; Eilers *et al.*, 1996; Quelle *et al.*, 1995; Valmas *et al.*, 2010). We found that, like the complete V protein, GFP–Vs co-precipitated both receptor-associated kinases, whilst the W domain precipitated neither (Fig. 2a, b). This interaction of GFP–Vs with Jak1 and Tyk2 was reflected in the ability of GFP–Vs to inhibit Jak1 and Tyk2 autophosphorylation, with the weaker co-precipitation of Tyk2 correlating with slightly weaker inhibition of this kinase (Fig. 2c, d). Interestingly, the W protein, although it did not co-precipitate either kinase, inhibited clearly Tyk2 autophosphorylation while having no detectable effect on Jak1 activity (Fig. 2c, d). We considered the possibility that this reflected the binding of W to STAT1, since there is clear evidence that STAT1 is a direct target of Tyk2, either when the kinase is expressed as a transgene (Eilers *et al.*, 1996) or when purified from IFN-treated cells (Usacheva *et al.*, 2003). STAT1 is also found in a complex with Tyk2 and RACK-1 (Usacheva *et al.*, 2003), and the binding of W to STAT1 might have been preventing the formation of the active Tyk2 phosphorylation complex as well as preventing the phosphorylation of STAT1 by preventing physically its association with RACK-1/Tyk2. We therefore created a mutant of W in which the tyrosine at aa 110 was converted to histidine (W_Y110H_), as this mutation has been shown to abolish binding to STAT1 in MV V protein (Devaux *et al.*, 2007). As expected, this mutant had lost the ability to co-precipitate STAT1 (Fig. 2e). However, the mutant W protein inhibited the autophosphorylation activity of Tyk2 as effectively as the native W protein (Fig. 2f), showing that it is not through STAT1 that the W protein is affecting Tyk2. The mechanism behind this particular activity of the P/V/W shared domain therefore remains to be determined.

**Fig. 3.**
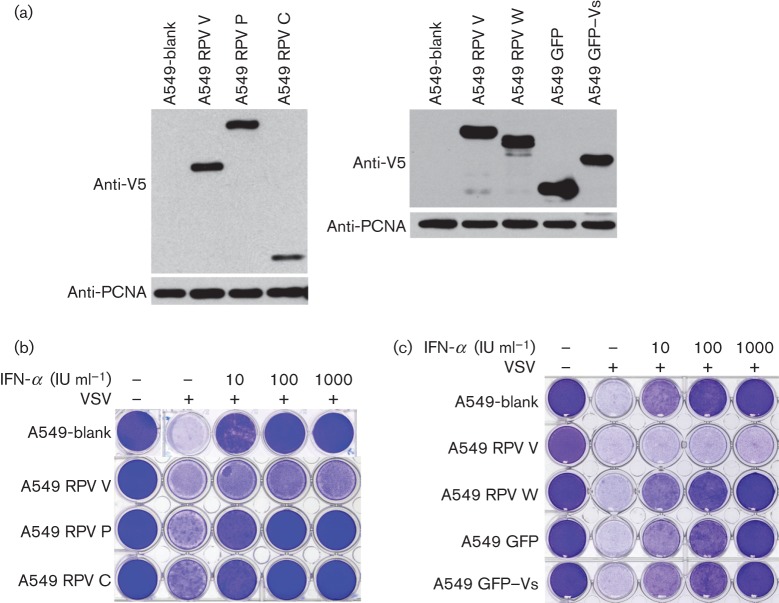
Inhibition of type I IFN action in stable cell lines. Stable cell lines transduced with lentivirus-based vectors encoding the RPV P, V, C or W proteins, or GFP or GFP–Vs were constructed as described in Methods; a cell line transduced with the base vector without an additional ORF (A549-blank) was used as a control. (a) The expression levels of the expressed viral proteins (or derivatives) were analysed by Western blotting using anti-V5 tag antibody; PCNA was used as the loading control. (b, c) Cell lines were plated on 24-well plates and treated for 24 h with the indicated concentration of IFN-α. The cells were then infected with VSV at an m.o.i. of 0.1. Cells were fixed 24 h post-infection and stained with crystal violet.

In order to test the actions of the P/V/W shared domain and the Vs domain in a possibly more biologically relevant assay, we also generated cell lines expressing these proteins and tested their abilities to block the induction of the antiviral state by type I IFN. The cells were treated with different concentrations of IFN-α and then challenged with vesicular stomatitis virus (VSV). The development of the antiviral state in the different cell lines is shown by the prevention of VSV-induced cytopathic effect and cell death. A cell line transduced stably with the empty vector (A549-blank) was generated to act as a negative control. Fig. 3(a) shows the expression levels of the viral proteins and derivatives in the stable cell lines. Comparison of cell lines expressing RPV C, P, V, W, GFP or GFP–Vs (Fig. 3b, c) showed that only the complete V protein could prevent the cells from entering the antiviral state. As expected from other studies, the C protein had no effect on type I IFN signalling; slightly unexpectedly, given their clear effects on IFN signalling as measured by the luciferase reporter gene assay, the P, W and GFP–Vs proteins all failed to block the induction of the antiviral state (Fig. 3b, c).

The physical separation of the RPV V protein into its N-terminal (W) and C-terminal (Vs) domains has shown that the V protein has at least two quite distinct methods of interfering with type I IFN action. We have also shown that neither functionality is sufficient on its own to enable the RPV V protein to show maximally efficient blocking of IFN signalling activity. The P/V/W shared domain, either on its own (as in the case of the W protein) or when present in the P protein along with the P-specific domain, was not sufficient to prevent cells entering the antiviral state in the functional assay, despite its ability to block IFN action when overexpressed in the reporter gene assay. Similarly, the Vs domain on its own showed a partial ability to block type I IFN action in the reporter gene assay, presumably due to its ability to bind to and inhibit the Jak1 and Tyk2 kinases, but could not prevent the function of type I IFN in inducing the antiviral state.

The data from our study on the RPV V protein are in agreement with previous studies with MV V protein, where both the N-terminal and C-terminal regions of the V protein were required for its efficient IFN signalling evasion activities, and the P/V/W shared domain and Vs domain on their own were shown to have only partial inhibitory effects on type I IFN signalling compared with the full-length MV V protein (Caignard *et al.*, 2007). A combination of mutations, Y110H in the P/V/W shared domain and C272R in the Vs domain, was shown to impair the IFN antagonistic activity of MV V protein (Ohno *et al.*, 2004), whilst another point mutation in the Vs domain of MV V protein, D248F, disrupted the ability of this protein to overcome the IFN-α-induced antiviral state, showing that the Vs domain is essential for proper V function (Ramachandran *et al.*, 2008).

Recent studies on the MV V protein have suggested that the P/V/W shared and Vs domains bind STAT1 and STAT2, respectively (Caignard *et al.*, 2007; Ramachandran *et al.*, 2008). Aa 110–130 in the MV V protein have been implicated in binding to STAT1 and a specific tyrosine residue at position 110 was shown to be critical in V–STAT1 interaction (Caignard *et al.*, 2007, 2009; Devaux *et al.*, 2007; Fontana *et al.*, 2008; Ohno *et al.*, 2004; Ramachandran *et al.*, 2008). Both the RPV V and W proteins used in this study had the Tyr110 residue and were able to co-precipitate STAT1. The MV Vs domain has been found to bind STAT2, and both a properly folded zinc finger domain and specific amino acids within the zinc finger domain were found to be required for this V protein to bind STAT2 (Caignard *et al.*, 2009; Ramachandran *et al.*, 2008). Interestingly, we only observed interaction of the RPV V protein with STAT2 when the Vs domain was present along with the P/V/W shared domain; the RPV Vs domain itself did not bind stably to STAT2. In this respect, the RPV V protein appears to resemble that of CDV (Rothlisberger *et al.*, 2010); when the Vs domain of CDV V protein was expressed fused to the HA epitope tag or to red fluorescence protein, the constructs did not co-precipitate STAT2, although the complete CDV V protein did. It is possible that the binding site for STAT2 on the morbillivirus V protein overlaps the point where the W and Vs domains meet, and small variations in the constructs used in the studies mentioned have led to the observed differences. The morbillivirus V proteins are clearly organized differently to those of the related henipaviruses, which seem to require only the N-terminal part of their V proteins for sequestering STAT1 and STAT2 in the cytoplasm, whilst the C-terminal region is totally dispensable for blocking IFN signalling (Rodriguez & Horvath, 2004).

The morbillivirus V proteins therefore have at least three functions that inhibit IFN signalling: the binding of STAT1 (seen also with the P and W proteins, and enabling the blockade of type II IFN signalling), the binding of STAT2 (requiring the Vs domain and part of the W domain), and the association with IFN receptor-associated kinases (requiring the Vs domain). All three activities combined form an extremely effective blockade of type I IFN signalling, rendering the infected cell essentially immune to the antiviral state.

## Methods

### 

#### Plasmids and other reagents.

Plasmids were cloned and grown in *Escherichia coli* (DH5α strain) and purified on CsCl gradients. The pcDNA constructs containing the P, C, W and V ORFs from the Saudi/81 strain of RPV, and the constructs encoding FLAG-tagged Jak1 and Tyk2, have been described previously (Boxer *et al.*, 2009; Chinnakannan *et al.*, 2013; Nanda & Baron, 2006). For all P, W and V expression constructs, modifications were made by overlap PCR mutagenesis to introduce three stop codons into the C ORF just after the C protein start codon, without altering the P/W/V ORFs, and a V5 epitope tag was added to the 5′ end of the coding sequence. The Vs domain was fused to EGFP (Clontech) by overlap PCR to generate pcDNA-GFP-Vs; pcDNA-GFP was constructed by amplifying just the EGFP ORF. All PCRs were performed using a proofreading polymerase (KOD; Novagen) and the PCR products introduced into plasmids were sequenced entirely. The pcDNA W_Y110H_ plasmid was created by site-directed mutagenesis using *Pfu* Turbo polymerase (Stratagene). The GFP–V–INS–Vs construct was based on the GFP–Vs construct, which was extended by an additional 12 aa (RSIASEKPIKKG) of the P/V/W shared region upstream of the Vs domain by site-directed mutagenesis. As a control, 12 non-V protein amino acids (VAYPYDVPDYAA; HA epitope tag) were added at the same position to create pcDNA-GFP-HA-INS-Vs. pCMYC-MDA5, pJAT-lacZ, pGAS-luc and the plasmid components of the lentivirus transduction system (pdlNotInPkMCSR, pMD-G and pCMVR8.91) were the kind gifts of Professor S. Goodbourn (St George’s Hospital Medical School, London, UK). pGL3-MX1P-luc was the kind gift of Professor Georg Kochs (Department of Virology, University of Freiburg, Germany). The plasmid encoding the PIV5 V protein (pEF-SV5V) was the kind gift of Professor R. E. Randall (University of St Andrews, UK).

Recombinant human IFN-αA was purchased from Calbiochem and human IFN-γ was purchased from Millipore. Sources of antibodies were: mouse anti-V5 (AbD Serotec), mouse anti-FLAG (M2 clone; Sigma), mouse anti-STAT1P (pY-701; BD Biosciences), rabbit anti-STAT1 (Upstate), rabbit anti-STAT2 (Upstate), rabbit anti-Tyk2P (pY-1054/1055; Cell Signalling Technology), rabbit anti-Jak1P (pY-1022/1023; Biosource International), rabbit anti-Tyk2 (Upstate), rabbit anti-Jak1 (Upstate) and mouse anti-PCNA (proliferating cell nuclear antigen; Santa Cruz Biotechnology). HRP-coupled anti-mouse or anti-rabbit antibodies used in Western blots were from GE Healthcare Life Sciences.

#### Cell lines and cell-based assays.

Derivation of stable cell lines expressing virus proteins was carried out using a pseudo-typed lentivirus-based vector system described previously (Demaison *et al.*, 2002; Hale *et al.*, 2006); other cell lines and their maintenance was as described by Chinnakannan *et al.* (2013). Cells were transfected with various plasmids using TransIT LT1 (Mirus) according to the manufacturer’s instructions. Luciferase assays and their statistical analysis, co-immunoprecipitation of STAT1/STAT2, and VSV cytopathic effect reduction assays on stable cell lines were carried out as described previously (Chinnakannan *et al.*, 2013). Co-immunoprecipitation of MDA-5 protein with viral proteins was assayed by transfection of 293FT cells with 1 µg pEF-cmyc-MDA5 together with 100 ng empty vector or plasmid expressing PIV5 V, RPV V, RPV W, GFP or GFP–Vs. At 48 h post-transfection, cells were lysed as for other immunoprecipitation studies and the lysates immunoextracted with anti-V5 mAb (Serotec). Immunoprecipitates were analysed by Western blotting using HRP-coupled mouse anti-c-myc antibody clone 9E10 (Roche).

Jak1/Tyk2 overexpression and co-immunoprecipitation assays were carried out using modifications of previously published methods (Chinnakannan *et al.*, 2013). Jak1/Tyk2 overexpression studies were carried out in Vero-SLAM cells and the amounts of each plasmid adjusted to give equivalent amounts of expressed protein. The following amounts of each construct were used for transfections: 400 ng pFLAG-Jak1 or 650 ng pFLAG-Tyk2 together with 75 ng pcDNA-GFP, 900 ng pcDNA-W, 1 µg pcDNA-V, 500 ng pcDNA-GFP–Vs or 1 µg empty vector (pcDNA6.1). The total amount of DNA was kept equal in all samples by adding empty vector where applicable. Cells were harvested 30 h post-transfection in SDS-PAGE sample buffer containing phosphatase inhibitors and analysed by Western blotting. Jak1 and Tyk2 co-immunoprecipitation assays were performed in 293FT cells, transfecting the following amounts of each construct: 1.2 µg pFLAG-Jak1 or 1.8 µg pFLAG-Tyk2 together with 200 ng pcDNA-GFP, 200 ng pcDNA-W, 250 ng pcDNA-V, 500 ng pcDNA-GFP–Vs or 500 ng pcDNA6.1. The total amount of DNA was kept equal in all samples by adding empty vector. Two days post-transfection, the cells were washed once with PBS, lysed and immunoextracted with anti-V5 mAb as described previously (Chinnakannan *et al.*, 2013).
